# Assessing the Progress towards Achieving “VISION 2020: The Right to Sight” Initiative in Ghana

**DOI:** 10.1155/2019/3813298

**Published:** 2019-07-22

**Authors:** Enyam Komla Amewuho Morny, Samuel Bert Boadi-Kusi, Stephen Ocansey, Samuel Kyei, Kwame Yeboah, Maureen Adanna Mmaduagwu

**Affiliations:** Department of Optometry and Vision Science, School of Allied Health Sciences, University of Cape Coast, Cape Coast, Ghana

## Abstract

**Purpose:**

The aim of this study was to analyse eye health delivery in Ghana and examine the progress towards achieving VISION 2020 indicator targets.

**Methods:**

This descriptive cross-sectional study was conducted between October 2017 and May 2018. It used a mixed method approach including desk-based reviews, a questionnaire-based survey of eye facilities in Ghana, and interviews with eye health system stakeholders to collect information on eye health delivery in facilities owned by the Ghana Health Service (GHS), quasigovernmental bodies (security agencies), and Christian Association of Ghana (CHAG). The information was benchmarked against the World Health Organization (WHO) targets for achieving the goals of VISION 2020.

**Results:**

The magnitude of blindness and moderate to severe visual impairment (without pinhole) was 0.9% and 3.0%, respectively. The number of ophthalmologists available at the country level was 80.6% of the VISION 2020 target with optometrists and ophthalmic nurses exceeding targets for VISION 2020. The distribution of human resources was heavily skewed towards two out of the 10 regions in Ghana. Cataract surgical rate was low and met 25% of the WHO target. Basic equipment for refraction was available in the majority of facilities; however, there was a general lack of specialised eye care equipment across the country. Comparatively, CHAG facilities were better equipped than GHS facilities at the same level.

**Conclusion:**

The Government of Ghana should revitalize the goals of VISION 2020 beyond the year 2020 and spearhead a concerted effort to ensure equitable distribution of human and infrastructural resources across the country.

## 1. Introduction

Visual impairment (VI) affects an estimated 442 million people globally, of whom 36 million are blind [1, 2]. Remarkably, the burden of visual impairment is unequally distributed as 90% of visually impaired people live in low- and middle-income countries (LMICs) [2]. Although 80% of VI including blindness is avoidable [1, 3], the number of people with VI is projected to rise to 700 million in 2050 [2] if adequate interventions are not implemented.

The World Health Organization (WHO) and the International Agency for the Prevention of Blindness (IAPB) launched the VISION 2020: the Right to Sight Initiative in 1999, to reduce avoidable VI and ultimately eliminate avoidable blindness by the year 2020 [4–6]. VISION 2020 focused on three major strategies: (i) disease control and eye care delivery, (ii) human resource development, and (iii) provision of appropriate technology and infrastructural facilities. These strategies were to be implemented at country levels through the development and implementation of individual national plans. The commitment of governments to the initiative was the single most important factor to achieving VISION 2020 [4].

Ghana signed on to the VISION 2020 programme by launching the National Eye Health Program on October 31^st^ 2000, and the country is believed to have made progress in reducing blindness and controlling eye diseases [7]. At the start of the initiative, 1% of Ghana's 18 million population were estimated to be blind and the leading causes of blindness were cataract, glaucoma, onchocerciasis, and corneal scarring [8]. There were 20 ophthalmologists, 75 ophthalmic nurses [8], and 50 optometrists [9], and 60% of all eye care personnel worked in two cities, Accra (Ghana's capital) and Kumasi (Ashanti Regional capital) [8]. With less than two years to go to the end of the programme, a comprehensive evaluation of the VISION 2020 initiative in Ghana is required to assess the progress towards achieving the goals of the initiative. The aim of the study was to evaluate the VISION 2020 initiative in government and Christian Health Association of Ghana (CHAG) owned facilities in Ghana. The information will be useful in the planning of eye care services beyond VISION 2020.

## 2. Methods

### 2.1. Study Setting

**Figure 1:**
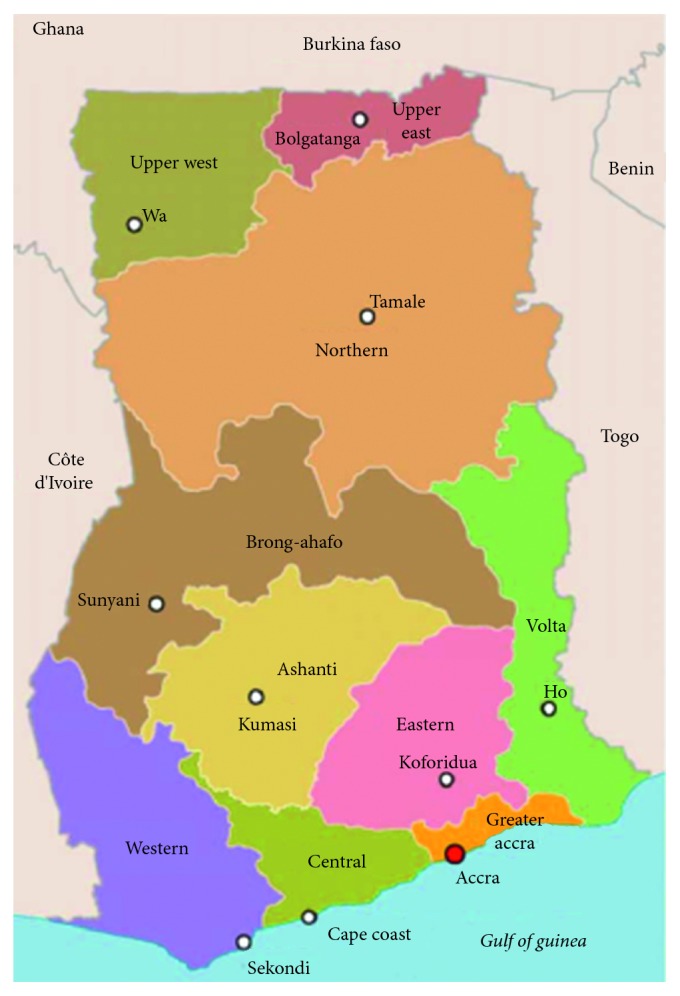
Map of Ghana showing the 10 administrative regions and their capital cities (available at https://goo.gl/images/8FwnbY; accessed 22/12/2018).

Ghana is a West African country with a population of 28.8 million in 2017. Nearly 40% of the population are under the age of 15 years [10]. The country is divided into 10 administrative regions (Figure 1) and subdivided into 212 districts. The Ministry of Health regulates all healthcare services and oversees the five major agencies through which health services are provided in Ghana [11, 12]. These agencies are the Ghana Health Service, teaching hospitals, quasigovernment hospitals (police and military), faith-based organizations (e.g., Christian Health Association of Ghana (CHAG)), and private clinics and maternity council. The first three agencies are state owned and are the main public sector agencies for healthcare delivery. The GHS is the largest service provider and operates facilities at the regional, district, subdistrict and community levels. CHAG is an autonomous nongovernmental agency comprising a network of 300 health facilities and 18 health training facilities owned by 25 different church denominations [13, 14]. It is the second largest health service provider. It works within the policies, guidelines, and strategies of the MOH and receives funding from government with 80% of CHAG's professional staff on government payroll [13]. This study surveyed only GHS, quasigovernment, and CHAG facilities with eye care units.

In this study, GHS facility respondents identified their facilities as regional, metropolitan, and municipal/district hospitals. The authors therefore adopted this categorisation. CHAG facilities are not stratified into levels; however, most of them operate at the district and subdistrict levels [12, 14]. The teaching hospitals and quasigovernment facilities were considered as tertiary facilities [15].

### 2.2. Data Collection

This descriptive cross-sectional study was conducted from October 2017 to May 2018 in accordance with the Declaration of Helsinki and was approved by the Institutional Review Board of the University of Cape Coast. Data were collected using a mixed methods approach from four sources: (i) desk-based review of institutional documents and publications, (ii) a questionnaire-based survey of eye facilities in Ghana using a questionnaire adopted from the Questionnaire on Available Human Resources, Infrastructure, and Equipment [16], (iii) interviews with eye health system stakeholders, and (iv) personal communication. The desk-based review and interviews were guided by the key indicators specified in the Catalogue of Key Eye Health Indicators in the African Region [17]. Triangulation was used to synthesise and integrate the data from different sources.

For the survey, a list of eye care facilities including their physical addresses and telephone numbers was first prepared with inputs from the National Eye Care Unit (NECU) of Ghana and the secretariats of the Ghana Ophthalmological Society (OSG), Ghana Optometric Association (GOA), and the Ophthalmic Nurses Group (ONG). The list was supplemented with information from eye care personnel who knew of other facilities not captured in the initial list. Private-for-profit eye care facilities were excluded. The survey questionnaire (supplementary data) was emailed to representatives at government and CHAG facilities for self-completion after a preliminary contact via email, telephone call, or text messaging or administered face-to-face during a visit to the facility. In addition, a link to an online version of the questionnaire, created using Google Forms©, was sent by text messaging or email to the representatives at the facilities. Reminders were sent via telephone calls or text messaging to maximize response. Data collected included background information on the facility, service delivery, human resources for eye health, and equipment.

Furthermore, the research team randomly selected and visited 15 of the responding facilities to validate the information collected. Data from the respondents were checked to avoid double entry. Information on the national level of eye care was collected in an interview with the Head of NECU, Dr. James Addy. The interview was conducted by two members of the research team (KY and MAM). Descriptive statistics were performed for the distribution characteristics of the human resource for districts and regions and infrastructure at the various facilities. VISION 2020 targets were used as a benchmark for data on human resource [18] while the IAPB Standard List of Equipment was used to benchmark equipment [19–21].

## 3. Results

### 3.1. Disease Control

The desk-based review of the literature identified one national survey of the prevalence of blindness, the Ghana National Blindness and Visual Impairment Study conducted in 2015 [22], as the most recent and robust nationally representative data on blindness and visual impairment in Ghana. Three other prior studies [23–25] on the prevalence of visual impairment and/or blindness in specific locations in Ghana were also identified (Table 1).

The national average prevalence of vision impairment (VA < 6/18) in Ghana was 5.0%. This includes the 0.9% of the population who were blind (VA < 3/60) [22] (Table 1). The principal causes of blindness (VA < 3/60) were cataract (54.8%), glaucoma (19.4%), posterior segment diseases (13.0%), and corneal opacity (11.0%). However, the five main causes of moderate to severe VI (MSVI, VA < 6/18 to 3/60) were refractive error (44.4%), cataract (42.2%), posterior segment diseases (9.0%), glaucoma (2.0%), and corneal opacity (2%).

#### 3.1.1. Cataract Surgical Rate and Refraction Rate

**Figure 2:**
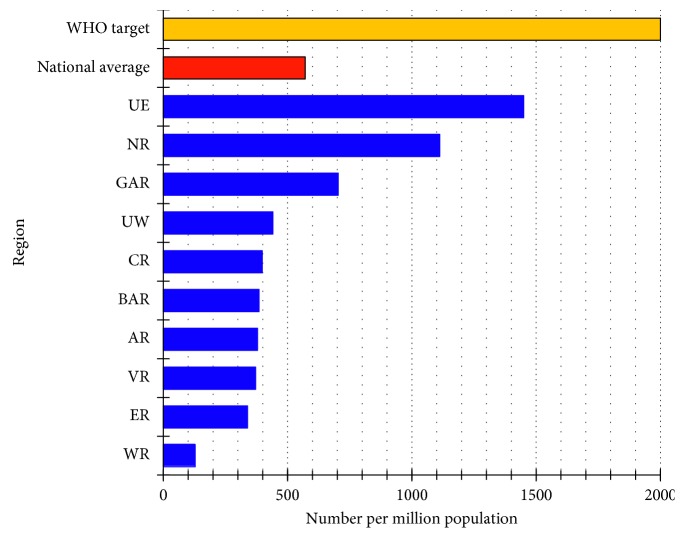
Cataract surgical rates in the 10 regions of Ghana compared to the WHO target in 2016. AR: Ashanti Region; BAR: Brong-Ahafo Region; CR: Central Region; ER: Eastern Region; GAR: Greater Accra Region; NR: Northern Region; UE: Upper East Region; UW: Upper West Region; VR: Volta Region; WR: Western Region.

The average national cataract surgical rate (CSR) in 2016 was 523 surgeries per million population [26], which fell short of the WHO target of 2,000 per million population. The Upper East had the highest CSR rate of 1,450 while the Western Region had the lowest rate of 128 (Figure 2).

Although many public facilities carried out refractions and dispensed spectacles, these were not routinely reported to the NECU. National refraction rates were therefore not available.

#### 3.1.2. Trachoma and Onchocerciasis

In June 2018, Ghana became the first country in the WHO African Region to eliminate trachoma as a public health problem under the Ministry of Health/Ghana Health Service led National Trachoma Elimination Programme. Onchocerciasis on the other hand remains endemic in 87 districts in Ghana despite long-term control with repeated ivermectin treatment [27].

### 3.2. Human Resource for Eye Health

**Figure 3:**
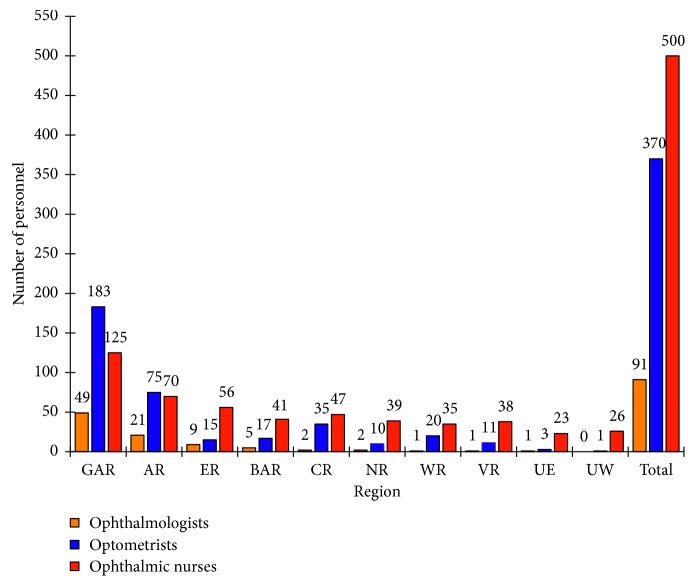
Number of eye health personnel in Ghana segregated according to regions in 2017. Abbreviations for regions are the same as in Figure 2. NB: number of optometrists includes private and public sector. Sources: NECU registers of ophthalmologists and ophthalmic nurses; GOA register of optometrists.

There were 91 ophthalmologists in Ghana in 2017 (NECU Register of Ophthalmologists) (Figure 3). The ophthalmologist to population ratio of 1 : 311,080 met 81% of the VISION 2020 target of 1 : 250,000. However, 78% of the ophthalmologists were based in Greater Accra Region (*n*=49) and Ashanti Region (*n*=21). On the other hand, the Northern Region had two ophthalmologists, the Upper East had one, and the Upper West Region had none.

There were 370 registered optometrists in Ghana (GOA Register of Optometrists) (Figure 3) representing an optometrist to population ratio of 1 : 76,508, lagging slightly behind the VISION 2020 target of 1 : 50,000 [18]. Again, 70% were based in Greater Accra (*n*=183) and Ashanti Region (*n*=75), while the Northern Region, Upper East Region, and Upper West Region had ten, three, and one, respectively.

There were 500 ophthalmic nurses at the country level (nurse to patient ratio = 1 : 56,616) with at least one ophthalmic nurse in every district (NECU Register of Ophthalmic Nurses), exceeding the recommended VISION 2020 target of 1 : 100,000.

### 3.3. Technology and Infrastructural Facilities

**Figure 4:**
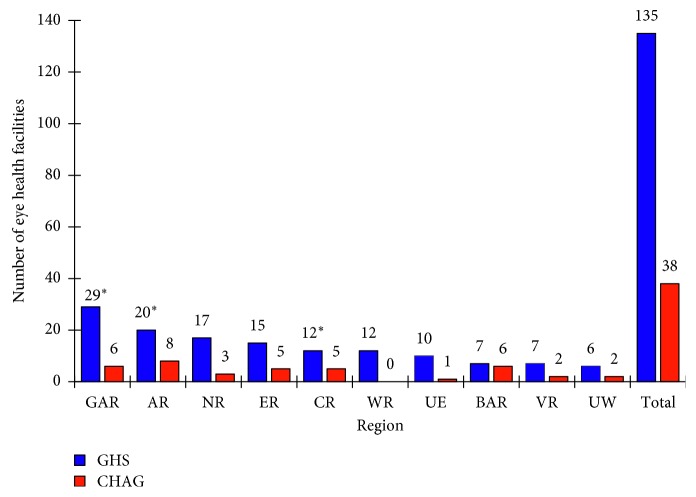
Number of eye health facilities in Ghana in 2017. CHAG: Christian Health Association Of Ghana; GHS: Ghana Health Service. Abbreviations for regions are the same as in Figure 2. Source: National Eye Care Unit. ^*∗*^The number of GHS facilities in GAR includes the one teaching hospital and two quasigovernment facilities. The number of GHS facilities in AR and CR includes one teaching hospital each.

This study identified 173 publicly funded facilities which provided eye care in Ghana: 133 GHS, 2 quasigovernment, and 38 CHAG owned (Figure 4). Greater Accra Region had the largest number of facilities (*n*=35), followed by Ashanti Region (*n*=27), with Upper West having the least number of facilities (*n*=8). An additional 61 privately owned facilities were identified, out of which 92% were located in Greater Accra (*n*=43) and Ashanti (*n*=14) regions (data not shown).

Questionnaires were sent to 148 facilities out of which 70 facilities responded (47.3% response rate). However, seven of the respondents were in private practice and were excluded in the analysis, leaving 63 facilities (42.6%) as eligible participants (Table 2). This was made up of 45 GHS facilities, two teaching hospitals, one quasigovernment facility, and 15 CHAG facilities.

Generally, basic equipment for refraction and the detection and management of cataract, glaucoma, and diabetic retinopathy was available in the majority of facilities (Table 3). For example, test charts, trial frames, and lens set were available in more than 90% of the facilities, while ocular health screening equipment like direct ophthalmoscopes and slit lamps were, respectively, available in 98% and 71% of the facilities (Table 3). However, advanced equipment was scarce. For example, the visual field analyser, a necessary equipment for the detection and monitoring of glaucoma, was available in eight out of 63 facilities surveyed. Furthermore, an optical coherence tomography (OCT) machine used in the management of glaucoma, diabetic retinopathy, and other retinal conditions was available in only one facility.

## 4. Discussion

The VISION 2020 programme was launched in 1999 with the twin aims of eliminating avoidable blindness by the year 2020 and preventing the projected doubling of avoidable visual impairment between 1990 and 2020 [6]. This study assessed the progress of Ghana in achieving targets set by the VISION 2020 initiative.

### 4.1. Disease Control

The causes of avoidable blindness and visual impairment that were targeted for elimination by the end of the programme were cataract, onchocerciasis, trachoma, childhood blindness, and refractive error [6]. The most current population-based survey conducted in 2015 put the prevalence of MSVI at 3% and blindness at 1% [22], similar to estimated prevalence rates in 2000, i.e., 3% and 1% for MSVI and blindness, respectively [28]. National Eye Health Programs in the last two decades focused mostly on adult eye care [26], a factor which may account for the stable prevalence rates. Furthermore, the introduction of the National Health Insurance Scheme (NHIS) in 2003 generally improved access to health care. Eye care services covered by the NHIS include consultation, cataract surgery, and refraction fees [29].

In absolute numbers, however, there was a 55% increase in the number of people with visual impairment and blindness, and these numbers are expected to increase due to population growth and increasing life expectancy. Nevertheless, the country has avoided the projected doubling of visually impaired and blind people in the country within the VISION 2020 project period.

Cataract remained the leading cause of avoidable and reversible blindness in Ghana, accounting for 40–60% of MSVI and blindness (Table 1). However, glaucoma was the leading cause of irreversible blindness. The disease was estimated to affect 7–15% of the population aged 30 years and above [30, 31]. Notably, the national CSR reached only 25% of the WHO target, although individual regional targets ranged from 5% in the Western Region to 75% in the Upper East Region (Figure 3). Factors contributing to the low CSR were poor access to eye care services (see section on facilities and equipment), inadequate human resources (especially ophthalmologists), and poor individual attitudes to the uptake of services. As an example, subdistricts and communities (where most of the rural population reside) receive eye care services through outreach programmes and special mobile eye clinics [32], yet a significant number of patients screened for cataract surgery do not report for surgery, whether on outreach services, eye camps, or within hospitals [33] due to costs and/or fear of surgery [34, 35].

Refractive errors were the leading cause of MSVI in Ghana accounting for 44% of all cases (Table 1). However, a study which assessed refractive error services (RES; refraction and dispensing of spectacles) provided by government and private facilities in the Northern and Central regions of Ghana found that the total RES output met 0.5–1.2% of the estimated demand [36]. Barriers to the uptake of RES included costs, being unaware of treatment options, and negative attitudes towards wearing spectacles [35, 37]. A study to integrate different school health interventions such as vision screening and deworming in a holistic and cost-effective manner [38] appears promising and would increase the provision of RES to school children. In addition, continued public education about the efficacy and safety of spectacles in correcting refractive error should be carried out by the Ministry of Health and its eye care providing agencies.

The WHO target of eliminating onchocerciasis by 2020 remained an arduous task in Ghana. The Onchocerciasis Control Programme (OCP) started in 1974 through vector control activities and later through the administration of ivermectin from 1987 [27, 39]. The 2017 Global Burden of Disease (GBD) Study reported a 93% reduction in the prevalence of onchocerciasis in Ghana from 2000 to 2017 [40]. However, an estimated 4.7 million people in 3,115 communities in 85 endemic districts from nine out of the ten regions remained at risk from the disease [41], with infection levels being 100 times the WHO threshold in some endemic communities [27]. Renewed and consistent efforts combining different methods of control will be needed to eliminate the disease [27, 41].

In June 2018, Ghana was declared trachoma free by the WHO and became the first country in the WHO Africa region to reach this goal (https://www.afro.who.int/news/ghana-eliminates-trachoma-freeing-millions-suffering-and-blindness; accessed 20/02/2018). Trachoma was endemic in the Upper West and Northern regions in the early 2000s [42, 43]. The key contributors to the success of the trachoma elimination programme were strong leadership at all levels especially at the national level, the implementation of the full SAFE strategy from the onset, and great collaboration between the GHS and partners in the programme [44].

The foregoing discussion suggests that the prevalence of visual impairment may further be reduced if programmes targeted at increasing CSR, providing refractive error and low vision services, and eliminating infectious causes of visual impairment are implemented. Data on the prevalence of childhood blindness in Ghana were scarce. It was estimated to account for 5% of blindness in the general population [45–47]. Specialist paediatric ophthalmology services were available only in two teaching hospitals, the Komfo Anokye Teaching Hospital, Kumasi, and Korle-Bu Teaching Hospital, Accra. This highlighted the importance of national eye care programmes for early childhood vision screening and establishment of paediatric eye care specialities in Ghana.

### 4.2. Human Resource for Eye Care

This study again documented the known inequitable distribution of human resources in Ghana [7, 48, 49]. The eye care workforce is concentrated in the southern and urbanised areas of Ghana (Figure 3), although the burden of vision loss is greater in the rural areas [22]. Ophthalmologists are medical doctors with specialised training in ophthalmic medicine and/or surgery and who evaluate and treat diseases of the eye [50]. Nearly all ophthalmologists are employed by the government to work in GHS or CHAG facilities. Although the current ophthalmologist to population ratio is 80% of the WHO target, many regions are poorly underserved.

Optometrists are primary healthcare practitioners who provide comprehensive eye and vision care, which includes refraction and dispensing, detection/diagnosis and management of disease in the eye, and the rehabilitation of conditions of the visual system. In many countries, including Ghana, optometrists are the first point of contact for persons with eye diseases [50]. Although, the optometrist to population ratio currently lags behind the WHO target by 35%, the number of optometrists in Ghana increased from less than 100 in 2000 to nearly 400 in 2017. Nevertheless, just about half of the optometrists (44%) are employed in the public and CHAG institutions with the rest in the private sector. Private eye care services are generally costlier than public services and often inaccessible to the poor especially in many developing countries [29].

Ghana is, however, on target for the number of ophthalmic nurses for eye care delivery. Currently over 90% of district hospitals have at least one eye nurse [12].

With the uneven distribution of workforce, the populace of regions with deficient workforce would have to travel longer distances for eye care. Also, with just few eye care personnel serving a large proportion of the populace, the patient load on these personnel would be extremely high, which would result in longer waiting time at the facilities, preventing many people from accessing essential eye care [51].

### 4.3. Technology and Infrastructural Facilities

This study identified 172 health facilities equipped at different levels to dispense eye care services. This represents 42% of the all public funded health facilities in Ghana of district level status and above [52]. Routine eye care service provision was unavailable at the subdistrict and community level facilities, which together make up 90% of all health facilities nationwide [52], as noted in previous studies [7, 12, 32]. The distribution of the eye care facilities was skewed to the Greater Accra Region where the proportion of the facilities exceeded the proportion of the population being served per WHO standards.

The majority of the facilities in each category were adequately equipped with basic equipment for refraction and funduscopy such as test types for distance and near, ophthalmoscopes, slit lamp biomicroscopes, and applanation tonometers. There was, however, a general unavailability of specialised equipment in many facilities, especially at the district level. This would impact negatively on the efficiency and quality of care provided by eye care staff [53]. For example, retinoscopes, autorefractors, and lensometers were unavailable in 50% or more of the metropolitan and municipal/district facilities (Table 3). Practitioners would therefore be limited to performing only subjective refraction.

Tertiary and regional hospitals were generally more equipped than metropolitan and municipal/district facilities. This limits the ability of these facilities to perform cataract surgery and also to predict a good prognosis of the cataract surgery. Field analysers for the early detection of glaucoma and OCT which helps to support the diagnosis of glaucoma and diabetic retinopathy were not available in any of the government-owned institutions (Table 3). Fundus cameras and argon lasers, which are useful for screening and managing retinal pathology including diabetic retinopathy, were available in only one tertiary facility and one regional hospital. This study further observed that CHAG facilities were better equipped than their counterpart GHS at the district/municipal level. For example, 80% of CHAG facilities had a slit lamp compared to 52% of GHS district/municipal facilities. Again, 73% of CHAG facilities had an applanation tonometer compared to 44% of the GHS facilities. This suggested that the CHAG facilities were better resourced to address the problem of visual impairment in Ghana.

## 5. Limitations of the Study

A component of this study was a questionnaire-based survey of eye care facilities in the country; less than 50% of departments returned a completed questionnaire. This may bias the results of the assessment of equipment towards an overestimation of how well departments are equipped and limit the extent to which the results may be generalised to the population. Furthermore, this study did not include private-for-profit facilities, which made up 26% of all eye care facilities in the country. Although these private facilities contribute actively to eye care services, especially RES [7, 36], 90% of the facilities were concentrated in two cities, i.e., Accra in the Greater Accra Region and Kumasi in the Ashanti Region. Their inclusion may bias the results towards an overestimation of refraction services and may not reflect the situation in most parts of the country.

## 6. Conclusion

Ghana has made bold strides towards the achievement of the VISION 2020 targets since its introduction in 2000. However, there remains an unequal burden of visual impairment in the country with rural communities bearing the greater share. Furthermore, the heavily skewed distribution of human resources for eye care, as well as technology and infrastructure, may continue to hinder the full realisation of the programme.

The Government of Ghana and the various stakeholders in eye care in Ghana need to revitalize the goals of VISION 2020 program beyond the year 2020. The new goals need to focus on strategies to ensure equitable distribution of personnel, equipment, and infrastructure across the country.

## Figures and Tables

**Table 1:** Population-based studies on prevalence of blindness in Ghana (presenting VA).

Study	Year	Location	Prevalence of MSVI and principal causes	Prevalence of blindness and its principal causes
Moll et al. [23]	1991	Wenchi	3.7%	1.7%
			Cataract (52%), refractive error (24%), nonglaucomotous optic atrophy (2%), vascular retinopathy (2%)	Cataract (63%), onchocerciasis (13%), corneal opacity (8%), refractive error (4%), phthisis (4%)
Guzek et al. [24]	2001	Ho, Hohoe, Kpando	15.0%	2.8%
			Cataract (54%), glaucoma (21%) and refractive error (17%), retinal disorders (9%); optic atrophy (8.8%), iatrogenic (3.9%)	
Budenz et al. [25]	2005	Tema	11.3%	1.2%
			Refractive error 61%, cataract 21%, glaucoma (5%), corneal opacities 3%, retinal disease 3%	Refractive error (35%), cataract (28%), glaucoma (14%), corneal opacities (6%), retinal disease (6%)
Wiafe et al. [22]	2015	Whole of Ghana	3.0%	0.9%
			Refractive error (44.4%), cataract (42.2%), posterior segment 8.9%, glaucoma (2.2%) and corneal opacities (2.2%)	Cataract (55%), glaucoma (19%), posterior segment diseases (13%), corneal opacities (11%)

MSVI: moderate to severe visual impairment, VA < 6/18 to 3/60.

**Table 2:** Distribution of eye care facilities who responded to the survey by regions in Ghana.

Region	Tertiary^*∗*^	Regional	Metro	Mun/Dist	CHAG	Total no. of facilities
Ashanti	—	1	2	1	3	7
Brong-Ahafo	—	—	—	1	2	3
Central	1	1	2	3	3	10
Eastern	—	—	1	5	1	7
Greater Accra	1	1	4	7	2	15
Northern	1	1	3	2	3	10
Upper East	—	—	—	1	—	1
Upper West	—	—	1	—	1	2
Volta	—	1	—	3	—	4
Western	—	1	1	2	—	4
Total	3	6	14	25	15	63

^*∗*^Comprises two teaching hospitals and 1 quasigovernment facility. Metro: metropolitan hospitals; Mun/Dist: municipal/district hospitals; CHAG: Christian Health Association of Ghana facilities.

**Table 3:** List of essential equipment available in the surveyed facilities.

Category of equipment	Listed equipment	Type of facility				
		Tertiary (*n*=3)	Regional (*n*=6)	Metro (*n*=14)	Mun/Dist (*n*=25)	CHAG (*n*=15)
Refraction	Autorefractor	1	2	4	3	3
	Lensometer	2	4	6	6	6
	Streak retinoscope	3	4	4	10	8
	Test type (distance)	3	6	13	23	15
	Test type (near)	3	6	12	23	15
	Trail frame	3	6	13	22	15
	Trial lens set	3	6	14	22	15

Cataract surgery	A-scan	1	1	1	0	1
	Applanation tonometer	2	6	8	11	11
	Autorefractor	1	2	4	3	3
	Binocular indirect ophthalmoscope	2	5	4	2	3
	Cataract surgical set	2	5	6	10	8
	Direct ophthalmoscope	3	6	14	25	14
	Gonio lens	2	1	2	0	2
	Head loupe	3	3	9	17	7
	Keratometer	2	1	2	2	4
	Operating microscope	3	5	3	6	6
	Slit lamp biomicroscope	3	6	11	13	12

Glaucoma screening, surgery and follow-up	A-scan	1	1	1	0	1
	Applanation tonometer	2	6	8	11	11
	ARGON laser	0	0	0	0	1
	Binocular indirect ophthalmoscope	2	5	4	2	3
	Direct ophthalmoscope	3	6	14	25	14
	Fundus camera	0	1	0	0	0
	Fundus lens	2	1	4	3	4
	Glaucoma set	1	1	3	1	3
	Gonio lens	2	1	2	0	2
	OCT	0	0	0	0	1
	Schiotz tonometer	2	3	10	13	7
	Slit lamp biomicroscope	3	4	11	13	12
	Visual field analyser	1	3	2	0	2
	YAG laser	1	1	0	0	2

Diabetic retinopathy screening	ARGON laser	0	0	0	0	1
	Binocular indirect ophthalmoscope	2	5	4	2	3
	Direct ophthalmoscope	3	6	14	25	14
	Fundus camera	1	1	0	0	0
	OCT	0	0	0	0	1
	Visual field analyser	1	3	2	0	2
	YAG laser	1	1	0	0	2

*n*: total number of facilities at each level.

## Data Availability

The reviewed data from previously reported studies and datasets supporting this study have been cited in this paper. The data used to support the findings of this study are available from the corresponding author upon request.

## Supplementary Materials

Supplementary Materials The supplementary data are a copy of the survey questionnaire used in the study.
