# Supplementary material for: Assessing the Progress towards Achieving “VISION 2020: The Right to Sight” Initiative in Ghana
Source: J Environ Public Health. 2019 Jul 22;2019:3813298. doi: 10.1155/2019/3813298 (PMC6679876; doi:10.1155/2019/3813298)
Supplement: Supplementary Materials — The supplementary data are a copy of the survey questionnaire used in the study. [file 3813298.f1.docx]

**UNIVERSIRTY OF CAPE COAST**

**DEPARTMENT OF OPTOMETRY**

**STUDY QUESTIONNAIRE**

**QUESTIONNAIRE ON AVAILABLE HUMAN RESOURCES, INFRASTRUCTURE AND EQUIPMENT**

| BACKGROUND INFORMATION | | |
| --- | --- | --- |
| Questions | Coding Categories | Code |
| Facility Tertiary Institutions……….............1  Specialist General Hospitals ...........2  General Hospital……………..........3  Primary Health Center (PHC)…......4  Health Clinic (HC)………………...5 | |  |
| Respondent Title: First Name: Surname:  …………………….. ………………………… ……………………… | | |
| Name of Facility Location (District, Region)  ………………………………………….. ………………………………………….. | | |
| D D M M Y Y | | |

HUMAN RESOURCE

Code for major role (indicate as many as applicable):

Surgery – 1; refraction – 2; vision screening – 3; assistance in surgery – 4; detection – 5; management of cataract and glaucoma – 6; low vision services – 7

| Cadre | Total number | Major roles |
| --- | --- | --- |
| Ophthalmologists (total) |  |  |
| Ophthalmologists - operating |  |  |
| Ophthalmologists – not operating |  |  |
|  |  |  |
| Optometrists |  |  |
|  |  |  |
| Full time eye worker – non doctor |  |  |
| Cataract surgeons |  |  |
| Orthoptists |  |  |
| Ophthalmic assistants |  |  |
| Ophthalmic nurses |  |  |
| Low vision specialist |  |  |
| Other mid-level eye care personnel |  |  |
|  |  |  |
| Primary Eye Care Workers |  |  |
| CDTI workers for onchocerciasis |  |  |
| Primary Health Care workers |  |  |
|  |  |  |
| Community workers |  |  |
| CBR workers |  |  |
|  |  |  |
| Allied eye health workers |  |  |
| Eye care managers |  |  |
| Equipment technician |  |  |
|  |  |  |
| Ophthalmologists operating on cataract |  |  |
| Eye care staff performing lid surgery |  |  |
| Eye care staff providing refraction services |  |  |

Maximum capacity:

Please indicate what you consider to be the maximum capacity per eye health worker per year under ideal conditions: (enough cases coming forward, sufficient support staff, sufficient facilities, equipment and supplies, but taking in consideration other tasks and responsibilities)

| Cataract operations in a year |  |
| --- | --- |
| Cataract operations per cataract surgeon per year |  |
|  |  |
| Trichiasis operations per lid surgeon per year |  |
|  |  |
| Low vision cases in a year |  |
| Low vision care per low vision specialist per year |  |
|  |  |
| Number of days/surgical cases |  |
| Number of surgeries per day |  |
|  |  |
| Number of patients seen/examiners |  |
|  |  |
| Number of spectacles dispensed/ optometrist |  |

INFRASTRUCTURE AND EQUIPMENT

| Instrument | No. available | No. in good working condition | No. beyond repair |
| --- | --- | --- | --- |
| Ophthalmic beds |  |  |  |
| Operating theatre |  |  |  |
| Operating tables |  |  |  |
| Outpatient department |  |  |  |
|  |  |  |  |
| Vehicle |  |  |  |
| Spare parts |  |  |  |
| Motorcycle |  |  |  |
| Bicycle |  |  |  |
|  |  |  |  |
| Slit lamp microscope |  |  |  |
| Applanation tonometer |  |  |  |
| Direct ophthalmoscope |  |  |  |
| Indirect ophthalmoscope |  |  |  |
| 20D lens |  |  |  |
| Goniolens |  |  |  |
| Fundus lens |  |  |  |
| Streak retinoscope |  |  |  |
| Binomag loupe with headband |  |  |  |
| Hand held slit lamp |  |  |  |
| Keratometer |  |  |  |
| Autorefractor |  |  |  |
| A-scan |  |  |  |
| B-scan |  |  |  |
| Field analyzer |  |  |  |
| Yag laser |  |  |  |
| Argon laser |  |  |  |
|  |  |  |  |
| Punctum dilator |  |  |  |
| Irrigation canula |  |  |  |
| Lid retractors |  |  |  |
| Schiotz tonometer |  |  |  |
| Head loupe |  |  |  |
| Hand held loupe |  |  |  |
|  |  |  |  |
| Trial lens set |  |  |  |
| Trial frames |  |  |  |
| Test types distance |  |  |  |
| Test types near |  |  |  |
| Lensometer |  |  |  |
| Torch |  |  |  |
| Examination loupe |  |  |  |
| Autoclave |  |  |  |
| Sterilizing drums |  |  |  |
| Eye pressure reducer |  |  |  |
| Drip stand |  |  |  |
| Operating lamp |  |  |  |
|  |  |  |  |
| Operating microscope |  |  |  |
| Assistant binocular microscope |  |  |  |
| Cataract surgery sets ECCE/ICCE |  |  |  |
| Glaucoma set |  |  |  |
|  |  |  |  |
| Lid surgery set |  |  |  |
| Chalazion set |  |  |  |
| Pterygium set |  |  |  |
| Enucleation / evisceration set |  |  |  |
| Cryo therapy |  |  |  |
| Bipolar cautery |  |  |  |
|  |  |  |  |
| Slide projector with trays |  |  |  |
| Tripod screen |  |  |  |
| Overhead projector |  |  |  |
| Transparencies and pens |  |  |  |
| Teaching slide sets |  |  |  |
| Library books |  |  |  |
|  |  |  |  |
| Suture materials |  |  |  |
| PC-IOL |  |  |  |
| AC-IOL |  |  |  |
| Artificial eyes |  |  |  |

| INDICATORS FOR TRENDS IN THE MAGNITUDE AND CAUSES OF VISUAL IMPAIRMENT | | | |
| --- | --- | --- | --- |
| Indicator | Absolute number | per 100 000 population | Data Source |
| What is the prevalence of visual impairment, including blindness, at country level? |  |  |  |
| What is the prevalence of blindness at country level? |  |  |  |
| What is the prevalence of visual impairment? |  |  |  |
| What are the number of districts in the country where blinding trachoma is a public health problem? |  |  |  |
| What are the number of onchocerciasis endemic communities identified and stratified? |  |  |  |

| HUMAN RESOURCES FOR EYE HEALTH | | | |
| --- | --- | --- | --- |
| Indicator | Absolute number | per 100 000 population | Data Source |
| What is the number of ophthalmologists active at country level? |  |  |  |
| What is the number of optometrists at country level? |  |  |  |
| What is the number of ophthalmic nurses at country level? |  |  |  |
| What is the number of opticians at country level? |  |  |  |
| Indicator | Proportion | | Data Source |
| What is the proportion of tertiary eye facilities with trained technicians? |  | |  |
| What is the proportion of secondary eye facilities with trained technicians? |  | |  |

| SERVICE DELIVERY | | |
| --- | --- | --- |
| Indicator | Absolute number | Data Source |
| What is the number of cataract surgeries performed in the previous year? |  |  |
| What is the cataract surgical rate at national level? |  |  |
| What is the number of cataract operated people with Visual Acuity >6/18 in the operated eye in the previous year? |  |  |
| What is the number of public health facilities at country level that dispense spectacles (glasses) for managing refractive errors? |  |  |
| What are the number of centers offering pediatric ophthalmology services in the country? |  |  |
| What is the number of centers providing laser treatment for diabetic retinopathy? |  |  |
| What is the number of centers which have a functioning visual field perimeter for glaucoma? |  |  |
| What is the number of patient consultations for eye diseases in the country per year |  |  |
| Does the national programme for Community Health Workers include eye health? |  |  |
| Indicator | Proportion | Data Source |
| What is the cataract surgical coverage at national level? |  |  |

| 5.0 HEALTH FINANCING | | |
| --- | --- | --- |
| Indicator |  | Data Source |
| Do budgetary appropriations for Eye Health exist at MoH level? | YES NO |  |

| CONSUMABLES AND TECHNOLOGY | | |
| --- | --- | --- |
| Indicator | Absolute number | Data Source |
| What is the number of eye care drugs included in the essential medicines list? |  |  |
| What is the proportion of all cataract operations performed with intraocular lens implantation in the previous year? |  |  |

NB: this part is not part of the questionnaire. However it contain some indicators that may be relevant to the study.

| TECHNOLOGY | | | |
| --- | --- | --- | --- |
|  |  |  | Y N F D |
| 0.1 | Glaucoma | Visual Acuity Charts with letters and tumbling Es  Ophthalmoscope  Tonometer  Perimeter  Slit Lamp  Slit Lamp Lenses – +90D & +60D or +78D  Applanation Tonometer attached to Slit Lamp  Gonioscopy  Pachymetry  Fundus Camera  Optical Coherence Tomography (OCT) |  |
|  | Low Vision and Refraction | Vision Screener test with Tumbling Es  Multiple Pinholes/Occluder  Confrontation test targets appropriate for use across the full range of acuities  LogMAR design Visual Acuity (VA) Chart  Contrast Threshold Tests with large targets  Autorefractor  Retinoscope  Retinoscopy lens rack  Prism Set  Lensmeter  Trial Lens Set  MAGNIFIERS – hand, stand, spectacle |  |

*Y – YES; *N – NO; *F – FUNCTIONAL; *D – NOT FUNCTIONAL
